# A rabbit model of ear otitis established using the *Malassezia pachydermatis* strain C23 from dogs

**DOI:** 10.14202/vetworld.2023.2192-2199

**Published:** 2023-11-01

**Authors:** Nadezhda Sachivkina, Arfenya Karamyan, Olesya Petrukhina, Olga Kuznetsova, Ekaterina Neborak, Alfia Ibragimova

**Affiliations:** 1 Department of Microbiology V.S. Kiktenko, Institute of Medicine, Peoples’ Friendship University of Russia (RUDN University), Moscow, Russia; 2 Department of Veterinary Medicine, Agrarian Technological Institute, Peoples’ Friendship University of Russia (RUDN University), Moscow, Russia; 3 Department of Biochemistry T.T. Berezov, Institute of Medicine, Peoples’ Friendship University of Russia (RUDN University), Moscow, Russia; 4 Department of Foreign Languages, Institute of Medicine, Peoples’ Friendship University of Russia (RUDN University), Moscow, Russia; 5 Department of General Pharmaceutical and Biomedical Technologies, Institute of Medicine, Peoples’ Friendship University of Russia (RUDN University), Moscow, Russia

**Keywords:** *in vivo* model, *Malassezia pachydermatis*, otitis media, rabbit

## Abstract

**Background and Aim:**

Fungal infections are a growing problem for both humans and animals due to the emergence of pathogenic strains resistant to modern antifungal treatments. To evaluate the efficacy of new antifungal drugs, it is essential to develop animal models that demonstrate typical responses to both the infection (pathogenesis and clinical course) and to the treatment, including adverse effects. In this study, we established a rabbit otitis model by infection of an aggressive multidrug-resistant strain from dogs, *Malassezia pachydermatis* C23, with no need for concomitant immunosuppression.

**Materials and Methods:**

Twenty healthy adult male gray giant rabbits (1 year old, 5.5 kg) were inoculated once with *M. pachydermatis* C23 at 10^8^ colony-forming units/mL. We observed the clinical signs of the disease and collected ear smears and blood samples every 5 days.

**Results:**

The infection progressed rapidly and exhibited characteristic clinical signs without spontaneous recovery for at least 1 month. In fact, substantial deterioration was observed as evidenced by blood parameters.

**Conclusion:**

This rabbit otitis model established using an aggressive drug-resistant fungus strain without immunosuppression could prove valuable for testing novel antifungal agents.

## Introduction

Otitis is a frequent reason for veterinary consultation, as approximately 20% of common household companion animals will have at least one otitis episode, and 3%–5% of dogs and cats will become chronically ill [1, 2]. Acute otitis externa is often suspected based on unusual behaviors in the home such as headshaking, ear scratching, rubbing against surrounding objects, and painful reactions to palpation of the auricles. In contrast, otitis media can be difficult to diagnose as afflicted animals are often asymptomatic unless the condition is associated with otitis externa or interna. The Basidiomycete yeast (BY) *Malassezia pachydermatis* is the most common cause of otitis media in animals [3, 4] and is usually isolated with pathogenic bacterial stains *Staphylococcus aureus*, *Enterobacterales* isolates, and *Pseudomonas aeruginosa* [5–7].

Despite the rich arsenal of treatments available for external ear inflammation, there has been a steady increase in the number of cases due to both adverse environmental changes and uncontrolled use of antimicrobial agents leading to dysbiosis and antibiotic resistance. Therefore, rational therapies for inflammatory diseases of the ear are urgently required in modern veterinary medicine.

The formation of biofilms contributes to the development of chronic infections [8–11]. Olabode *et al*. [3] isolated the aggressive *M. pachydermatis* strain C23, that demonstrated extensive biofilm production and strong resistance to antimycotic drugs. It was suggested during the manuscript review process that we examine the effects of *M. pachydermatis* C23 infection on laboratory animals. Among the most convenient vertebrate models for *Malassezia* infection is the immunocompromised, Swiss mouse proposed by Schlemmer *et al*. [12]. These mice were first immunosuppressed with a combination of cyclophosphamide at 150 mg/kg and hydrocortisone acetate at 250 mg/kg as infection did not develop in immunocompetent mice. Two separate groups were then established according to infection site, a dermatitis group receiving intradermal injection of 5 × 10^6^ fungal cells/mouse at a shaved dorsal region, and an otitis group receiving the same inoculum in the middle ear. However, in both groups, the presence of BY decreased over time and was undetectable on the 17^th^ day after intradermal injection and the 21^st^ day after middle ear inoculation. Therefore, this is not the most advantageous model to assess new treatment approaches. Furthermore, although invertebrate models have been established in *Drosophila melanogaster* [13] and *Caenorhabditis elegans* [14], these do not recapitulate the disease course in common companion animals.

In this study, we developed *M. pachydermatis* infection model that progresses rapidly, does not require immunosuppression or the use of other drugs, and allows for objective assessment of the clinical course using standard smears and blood chemistry.

## Materials and Methods

### Ethical approval

All animal experiments were performed in accordance with the Guide for the Care and Use of Laboratory Animals [15] and were approved by the Ethics Committee for Animal Experimentation, Peoples’ Friendship University of Russia, Moscow, Russia (protocol number 75, date: May 10, 2023).

### Study period and location

The study was conducted from April to June 2023. All experiments were performed at the Department of Veterinary Medicine, Agrarian Technological Institute, Peoples’ Friendship University of Russia (RUDN University), Moscow, Russia.

### Animals

**Figure-1:**
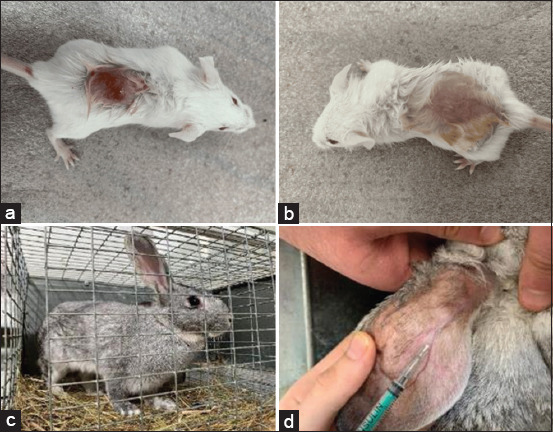
Stages of preparation of the experiment on mice (a and b) and rabbits (c and d) of the gray giant breed.

Animal models of disease should be established using the lowest strata that still recapitulate the typical pathogenesis and treatment response. Initially, we attempted to induce *Malassezia* infection in mice without reducing immunity but found the auricles too delicate to make incisions and too small to observe infection development. In addition, mice immediately lapped up the fungal suspension. Alternatively, a flat area was shaved on the back and 0.5 mL of fungal suspension was applied. The mouse was then hand-held for a few minutes to prevent licking the inoculum before being released back into the cage for observation. However, signs of dermatitis were still not observed within 1 week. It is believed that the course of infection may depend on the hormonal status of the individuals taken in the experiment. In this regard, we synchronized all animals for this trait by stimulating estrus by administering 25 mg/kg estradiol (Mesalin, Intervet Schering-Plough Animal Health, the Netherlands) to female mice. In this case, Malassezious dermatitis of the back was observed within 3 days but lasted only 7–20 days without additional treatment (Figure-1a and b). Furthermore, mice were able to physically remove therapeutic solutions. Therefore, it was decided to establish a model of ear otitis in rabbits.

Twenty healthy adult male gray giant rabbits (1 year old, weighing 5.5 kg) were used in the experiment (Figure-1c and d). Animals passed veterinary control, had all relevant documentation, and remained under the care of a veterinarian (Dr. A. Karamyan) during the entire experimental period. Rabbits were housed in 50.0 cm × 40.0 cm × 30.0 cm (W × L × H) cages under controlled ambient temperature (20°C ± 1°C) and the relative humidity (45% ± 1%) with food and water provided *ad libitum*. The granular feed included meadow and mountain herbs, apple pomace, sunflower seed flour, flax seed, linseed oil, oligosaccharide fructans, dried cucumber, dried carrots, dried celery, dried zucchini, calendula flowers, red clover flowers, hop cones, yeast extract, and yucca extract.

### Infectious strain

All strains of BY *Malassezia* were stored at −80°C. Stain *M. pachydermatis* C23 was chosen for model development because, in a study by Olabode *et al*. [3], it was the strongest biofilm producer (optical density = 0.441 ± 0.016) and most resistant to antifungal drugs among those tested. After thawing, samples were cultured on Sabouraud dextrose agar (SDA, Difco, Bordeaux, France) at 37°C for 72 h. According to Kirby–Bauer’s disk diffusion method, the strain possesses high resistance to amphotericin B, ketoconazole, and fluconazole, intermediate resistance to nystatin, clotrimazole, voriconazole, and itraconazole, and sensitivity to miconazole (HiMedia™ Laboratories Pvt. Ltd., Mumbai, India). After 3 days of incubation on agar, cultures were washed twice with sterile Brain Heart Infusion Broth (HiMedia™ Laboratories Pvt. Ltd.,), and suspended at McFarland 0.5 using a DEN1 McFarland Densitometer (Grant-bio, Grant Instruments Ltd., Cambridge, UK) for administration [16].

### Experimental infection of rabbits

**Figure-2:**
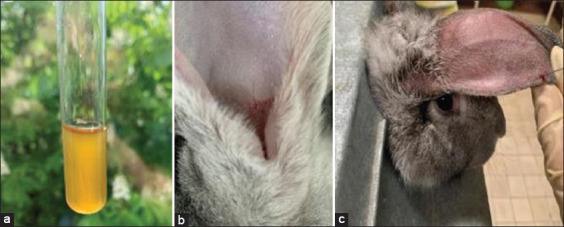
Experimental stages: (a) Preparing basidiomycete yeast suspension in Brain Heart Infusion Broth, (b) scratches on the ear, and (c) rabbit fixation and blood draw.

Rabbits were infected by topical administration of 1 mL BY suspension (10^8^ colony forming unit/mL) onto both auricles prescratched with a sterile blade (Figure-2). Animals were observed daily thereafter. No other treatments, including immunosuppressive drugs, were administered.

### Clinical signs and microbiological analysis

Clinical signs such as loss of appetite, decreased body weight, erythema, itching, and increased cerumen secretion (yellow-brown in color and often with an unpleasant odor) were rated from mild to severe and recorded.

Ear smears were acquired every 5 days, examined by light microscopy, and cultured on nutrient medium. *Malassezia pachydermatis* was found in all smears as confirmed by growth on SDA. Blood samples (3 mL) were also collected from the marginal ear vein before and during infection for measurement of hematological, biochemical, and immunological parameters using conventional methods.

### Blood and serum analysis

Red blood cell count, hemoglobin concentration, hematocrit, total white blood cell count, and individual white blood cell counts were measured using a hematological analyzer [17]. Serum was isolated for measurement of liver function enzymes such as alanine aminotransferase (ALT) and alkaline phosphate (ALP), and serum was isolated for measurement of glucose, urea, creatinine, calcium, albumin, total protein, cholesterol, triglycerides, and uric acid using a Mindray BC-2800Vet analyzer (Mindray, China), Chem Analyzer model BTS (BioSystem, Spain), or a diagnostic kit (BioMed Diagnostics, White City, Oregon, USA) according to manufacturer’s instructions.

### Statistical analysis

All results are expressed as mean ± standard error of mean. Means were compared by paired-sample t-test and p ≤ 0.05 was considered significant for all tests, and all statistical calculations were performed using XLSTAT 2020 software (Lumivero, Denver, CO, USA). All graphs were plotted using Microsoft Excel (Microsoft Excel for Office 365 MSO, Microsoft, Redmond, WA, USA).

## Results

### Course of experimental infection in rabbits

**Figure-3:**
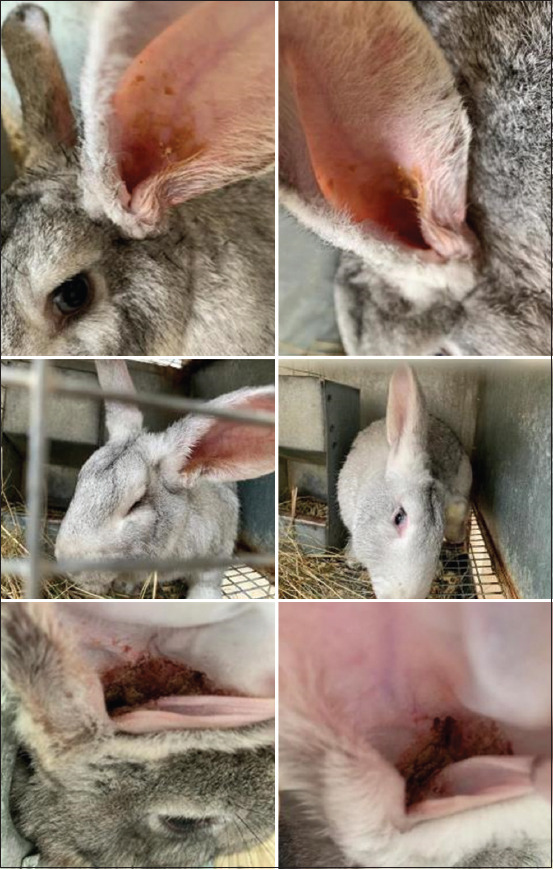
Clinical signs of *Malassezia otitis* media.

By the 3^rd^ day after inoculation, redness appeared on the inner surface of the ears. Between days 5 and 7 post-inoculation, a scab-like plaque formed on the skin in the depths of the auricles, and animals exhibited behavioral signs of severe itching. On day 10, the rabbits were observed shaking their heads and scratching their ears, and a significant increase in scabs was observed in the auricles. In addition, both activity and appetite were reduced. On palpation of the ears, local temperature and sensitivity increased. The clinical course of experimental otitis media is illustrated in Figure-3 and summarized by day in Table-1. At the beginning of infection, otoscopy revealed no detectable changes in the walls of the auditory canal and no pathological discharge was noted. Furthermore, the tympanic membrane was clearly visible. Starting from day 15, however, narrowing of external auditory canal due to constriction of the walls and abundant viscous brown discharge were observed, and the tympanic membrane was no longer visible.

**Table-1:** Severity of different clinical signs in rabbits’ model of *Malassezia* otitis media.

Parameters	Control	5 days experiment	10 days experiment	15 days experiment	20 days experiment	25 days experiment	30 days experiment
Loss of appetite or body weight	−	−	−	+	+	+	++
Erythema	−	+	++	++	+++	++++	++++
Itching	−	−	+	++	+++	++++	++++
Cerumen	−	−	+	+	++	++++	++++
Unpleasant odor	−	−	−	+	++	++++	++++

−=Normal, +=Mild, ++=Moderate, +++=Severe, ++++=Very severe

### Microbial analysis

Ear swabs were taken every 5 days and both examined by microscopy and inoculated into nutrient medium for microbial analysis. *M. pachydermatis* was detected within 5 days post-inoculation under the microscope and confirmed by growth on SDA. We also quantified changes in the number and composition of other microorganisms in the ear canal (Table-2) following protocols similar to those used by Sachivkina *et al*. [16] on changes in the species and quantitative composition of microorganisms in poultry with experimental candidiasis.

**Table-2:** Microbiological assessment of the *Malassezia* otitis media development in rabbits.

Parameters	Control	5 days experiment	10 days experiment	15 days experiment	20 days experiment	25 days experiment	30 days experiment
Presence BY in smears of contents from the ears	−	+	+	+	++	++	+++
Growth *Malassezia pachydermatis* on nutrient media	−	+	++	++	+++	++++	++++

−=Absence, +=Minor, ++=Moderate, +++=Abundant, ++++=Massive. BY=Basidiomycete yeast

### Changes in hematological parameters

Hematological analyses (Table-3) revealed modest increases in the total number of leukocytes (total WalsBC), granulocytes, neutrophils, and platelets in the few days post-inoculation, and more rapid increases thereafter. In contrast, erythrocyte count, hemoglobin concentration, hematocrit, average volume of erythrocytes (mean corpuscular hemoglobin [MCH]), average concentration of corpuscular hemoglobin (MCH concentration), lymphocyte count, and monocyte count decreased below baseline levels during the month post-inoculation (p < 0.05).

**Table-3:** Hematological parameters in rabbits’ model of *Malassezia* otitis media.

Parameters	Control	5 days experiment	10 days experiment	15 days experiment	20 days experiment	25 days experiment	30 days experiment
Total white blood cells (10^3^/µL)	4.71 ± 0.65	4.93 ± 0.71	5.03 ± 0.53	5.11 ± 0.47	5.25 ± 0.56	5.74 ± 0.62	6.23 ± 0.38*
Lymphocytes (10^3^/µL)	3.24 ± 0.60	3.11 ± 0.34	2.90 ± 0.27	2.96 ± 0.48	2.70 ± 0.42	2.72 ± 0.41	2.61 ± 0.44
Neutrophils (10^3^/µL)	2.03 ± 0.48	2.18 ± 0.63	1.99 ± 0.36	2.10 ± 0.53	2.24 ± 0.51	2.20 ± 0.49	2.28 ± 0.35
Monocytes (10^3^/µL)	0.39 ± 0.10	0.40 ± 0.13	0.42 ± 0.11	0.40 ± 0.13	0.45 ± 0.15	0.50 ± 0.16	0.50 ± 0.11
Granulocytes (10^3^/µL)	2.23 ± 0.29	3.02 ± 0.49	3.13 ± 0.35*	3.08 ± 0.28*	3.23 ± 0.40*	3.18 ± 0.32*	3.43 ± 0.55*
Red blood cells (10^6^/µL)	5.22 ± 0.46	5.50 ± 0.32	5.44 ± 0.42	5.59 ± 0.51	5.61 ± 0.43	5.52 ± 0.49	5.62 ± 0.38
Hemoglobin (g/dL)	10.66 ± 0.49	10.03 ± 1.15	9.42 ± 0.91	9.43 ± 1.21	9.25 ± 1.30	8.73 ± 0.94*	9.05 ± 1.17*
Hematocrit (%)	40.03 ± 1.90	35.19 ± 3.23	36.33 ± 4.11	34.92 ± 3.87	35.04 ± 4.25	34.71 ± 3.86	33.20 ± 4.73*
Mean corpuscular volume (fL)	62.27 ± 2.17	65.32 ± 2.53	66.77 ± 1.93	68.01 ± 2.68*	67.84 ± 3.03*	68.35 ± 2.63*	67.92 ± 2.96*
Mean corpuscular hemoglobin (pg)	21.91 ± 1.35	19.68 ± 0.94*	19.80 ± 1.25*	20.00 ± 1.04*	19.26 ± 0.92*	19.81 ± 0.95*	18.60 ± 0.98*
Mean corpuscular hemoglobin concentration (g/dL)	40.53 ± 1.02	38.64 ± 1.13	36.94 ± 1.54	32.51 ± 1.27*	30.60 ± 2.02*	31.81 ± 1.86*	30.40 ± 2.18*
Platelets (10^3^/µL)	190.73 ± 14.20	201.50 ± 25.72	209.58 ± 27.43	212.60 ± 23.04	211.94 ± 20.72	221.29 ± 25.05	230.74 ± 25.22

The data are represented as mean ± standard deviation.

* Statistically significant difference between experience and control (p < 0.05)

### Changes in biochemical parameters

Concentrations of high-density lipoproteins, cholesterol, and triglycerides increased significantly after experimental infection, while blood sugar and plasma fibrinogen levels decreased below baseline (p < 0.05) (Table-4). Other indicators, including low-density lipoproteins, urea, uric acid, creatine, calcium, serum globulin, albumin and total protein, were not significantly changed by infection.

**Table-4:** The biochemical parameters in rabbits’ model of *Malassezia* otitis media.

Parameters	Control	5 days experiment	10 days experiment	15 days experiment	20 days experiment	25 days experiment	30 days experiment
Blood sugar	120.77 ± 4.81	105.38 ± 4.02*	103.00 ± 5.93*	108.32 ± 4.74*	106.88 ± 4.81*	105.61 ± 4.60*	104.02 ± 5.09*
Cholesterol	118.36 ± 2.75	127.25 ± 3.81*	128.82 ± 4.01*	126.15 ± 4.78*	128.25 ± 3.27*	127.91 ± 5.03*	127.90 ± 4.62*
Triglyceride	138.62 ± 4.23	149.07 ± 3.90*	150.84 ± 3.52*	148.11 ± 3.46*	147.90 ± 4.32*	151.87 ± 4.73*	150.28 ± 4.92*
Uric acid	1.73 ± 0.39	1.96 ± 0.35	2.01 ± 0.40	1.98 ± 0.37	2.00 ± 0.42	1.87 ± 0.55	1.93 ± 0.50
Urea	30.90 ± 4.13	33.61 ± 5.17	32.74 ± 5.91	33.00 ± 4.47	34.58 ± 4.90	33.04 ± 5.88	34.23 ± 4.65
Creatine	0.75 ± 0.14	0.80 ± 0.13	0.80 ± 0.13	0.80 ± 0.13	0.80 ± 0.13	0.80 ± 0.13	0.80 ± 0.13
Calcium	9.45 ± 1.80	10.63 ± 1.44	10.26 ± 1.82	11.03 ± 2.05	10.43 ± 1.91	10.52 ± 1.04	10.37 ± 1.68
Albumin	5.44 ± 0.83	4.36 ± 1.25	5.03 ± 1.74	4.93 ± 1.62	5.06 ± 1.24	4.99 ± 2.23	4.81 ± 1.76
Total protein	7.60 ± 1.33	8.42 ± 1.28	6.90 ± 2.04	8.36 ± 1.31	8.53 ± 1.83	7.52 ± 1.75	8.04 ± 1.80
High density lipoprotein (mg/dL)	40.26 ± 3.05	49.10 ± 2.97*	45.83 ± 2.25	46.03 ± 2.40	49.13 ± 2.20*	47.99 ± 1.94*	50.08 ± 1.96*
Low density lipoprotein (mg/dL)	14.10 ± 1.12	15.17 ± 1.48	16.32 ± 2.01	15.66 ± 1.29	16.03 ± 1.41	16.82 ± 1.27	17.02 ± 2.04
Plasma fibrinogen	610.1 ± 303.7	464.7 ± 235.7*	464.7 ± 235.7*	464.7 ± 235.7*	464.7 ± 235.7*	464.7 ± 235.7*	464.7 ± 235.7*
Serum globulins	2.36 ± 0.43	2.80 ± 0.82	2.74 ± 0.51	2.68 ± 0.63	2.63 ± 0.39	2.70 ± 0.48	2.51 ± 0.36

The data are represented as mean ± standard deviation.

* Statistically significant difference between experience and control (p < 0.05)

### Changes in liver function enzymes

Serum concentrations of ALT, ALP, bilirubin, aspartate transaminase, and lactate dehydrogenase increased following experimental infection (p < 0.05) (Table-5).

**Table-5:** Liver function enzymes in rabbits’ model of *Malassezia* otitis media.

Parameters	15.537 pt	5 days experiment	10 days experiment	15 days experiment	20 days experiment	25 days experiment	30 days experiment
Alanine aminotransferase (IU/L)	113.56 ± 5.80	123.07 ± 6.18	121.38 ± 5.82	129.37 ± 4.99*	130.05 ± 6.16*	127.83 ± 5.84*	131.86 ± 6.01*
Alkaline phosphatase (IU/L)	199.05 ± 12.86	230.46 ± 15.05*	228.71 ± 9.42*	231.88 ± 10.92*	226.76 ± 11.51*	235.04 ± 10.83*	239.67 ± 9.59*
Bilirubin (mg/dL)	0.50 ± 0.04	0.59 ± 0.03*	0.60 ± 0.05*	0.58 ± 0.03*	0.58 ± 0.03*	0.61 ± 0.04*	0.59 ± 0.03*
Aspartate transaminase (IU/L)	95.00 ± 10.60	127.94 ± 8.30*	131.17 ± 14.65*	129.36 ± 12.58*	128.53 ± 9.58*	130.04 ± 11.74*	126.00 ± 10.31*
Lactate dehydrogenase (IU/L)	420.20 ± 36.51	509.63 ± 48.50	495.10 ± 38.50	510.93 ± 41.32*	519.40 ± 38.65*	512.94 ± 39.66*	515.70 ± 42.23*

The data are represented as mean ± standard deviation.

* Statistically significant difference between experience and control (p < 0.05)

## Discussion

The emergence of multidrug-resistant fungi has necessitated new treatment approaches, including the development of novel antifungal drugs and combination treatments such as the use of plant extracts to enhance the effects of conventional antimycotics. This process in turn requires the establishment of reliable animal models to assess treatment safety (toxicity) and efficacy [18–22].

Such models have been established using mice, rats, guinea pigs, dogs, and rabbits [23–25]. While these models have enabled the identification of various virulence factors for pathogenic fungi such as *Candida albicans* [26], they have been unsuccessful in modeling *Malassezia* infection due to the low virulence of many species within this genus. The first attempts to develop a suitable model for *Malassezia* spp. were unsuccessful due to natural recovery, indicating that the healthy immune system can effectively cope with these pathogens. In 1940, Moore *et al*. [27] exposed four species to *Malassezia furfur*, rabbits, guinea pigs, rats, and mice, by direct application on the skin but found no signs of infection, while intradermal or intratesticular inoculation caused long-term infection. It is also possible to infect guinea pigs with *M. furfur*, but this requires daily direct inoculation on intact skin for at least 7 days [28]. Similar results were found for *Malassezia restricta* when inoculated directly onto the skin of guinea pigs, with severe inflammation resembling seborrheic dermatitis observed after repeated inoculation every 24 h for 7 days and lasting 52 days. This model has been used to evaluate the antifungal activity of two drugs [29]. In 1986, several experimental infection models were developed, including dog, guinea pig, and rabbit models, to study the antifungal properties of killer yeast toxins such as *Hansenula anomala* UCSC 25F. In these experiments, cutaneous application of *M. furfur* was used to induce seborrheic dermatitis while auricular application of *M. pachydermatis* was used to induce otitis externa, and clinical recovery following killer toxin treatment was confirmed by the absence of BY cultures in smears [30].

In 1992, eight beagle dogs were experimentally injected with *M. pachydermatis* into the auricle to induce acute otitis externa and examine the efficacy of antifungal drugs. After 3–4 days, the animals showed the first symptoms of otitis externa. The entire ear canal was red, and the dogs were constantly shaking their heads and scratching their ears with their paws. Daily examination of ear exudate revealed high levels of *M. pachydermatis* in cultures [31]. Similar experiments were conducted on rabbits directly inoculated on the skin surface, with or without coverage with plastic sheeting to promote colonization. These treatments resulted in the appearance of skin lesions and mycelial structures in histological samples only in cases where the administration site was covered. However, spontaneous healing occurred if inoculation was terminated [32, 33].

Predisposing factors for otitis and dermatitis include an impaired skin barrier. Sparber *et al*. [34] reported that experimental percutaneous *Malassezia* spp. infection can be achieved by first breaking the dorsal skin of the mouse ear with adhesive tape. This study also showed that *Malassezia* exposure induced the release of interleukin-17, which in turn stimulated atopic dermatitis.

The emergence of drug-resistant microorganisms has increased exponentially despite the discovery of new therapeutic antibiotics [35–40]. Identifying new phytopreparations that can destroy single planktonic cells and microbial communities in biofilms, thereby preventing antibiotic resistance, an important development [13, 41–46]. In parallel with these discoveries, it is also essential to develop *M. pachydermatis* infection models that are reproducible, inexpensive, recapitulate the important pathological features of infection, and allow for objective assessment of both clinical course and treatment response.

## Conclusion

We developed a new animal model for drug-resistant *M. pachydermatis* infection that required no immunosuppression and showed little spontaneous remission. Rabbits were infected with the highly aggressive *M. pachydermatis* strain C23 by direct application on pre-abraded rabbit auricle. Infection progressed rapidly, as evidenced by characteristic clinical signs and changes in various hematological parameters. Future experiments are required to assess the sensitivity of this model to various antifungal medications and adjuvants such as Farnezol. Such experiments could also help elucidate the antimicrobial mechanisms of quorum-sensing molecules against *M. pachydermatis* and other biofilm-producing pathogens.

## Authors’ Contributions

NS and AK: The original idea for the study and designed the study. OP and AI: Collected the samples. OK and EN: Data analysis and data cleaning. NS and AI: Drafted the manuscript. All authors have revised the manuscript. All authors have read, reviewed, and approved the final manuscript.
